# A simple reproducible method of preventing lobar torsion

**DOI:** 10.1186/1749-8090-3-22

**Published:** 2008-04-30

**Authors:** Manoj Purohit, Joseph Zacharias

**Affiliations:** 1 Department of Cardiothoracic Surgery, Lancashire Cardiac Centre, Blackpool Victoria Hospital, Blackpool, UK

## Abstract

Torsion of remaining lobes after pulmonary resection is a potentially serious complication with high reported morbidity and mortality. A range of additional procedures has been described, we have used a simple, effective, quick and easy to reproduce minor procedure to prevents torsion.

## Review

Torsion of one of the remaining lobes after lung resection is a rare but potentially serious complication with disastrous consequences if unnoticed. A recent large series from Mayo clinic has documented an incidence of 0.089% after lung resection, 12% to 16% mortality has been reported for complicated torsion[1,2]. We have found this a particular concern after completing a right upper lobectomy, as the middle lobe is very likely to tort on its vascular pedicle, especially when the oblique fissure is well developed. This potential complication has been suspected, diagnosed and managed since the early days of lung resection and there are many methods described in the literature for fixing the remaining lobes to prevent lung torsion [2,3].

We describe a novel method of fixing the right middle lobe after right upper lobectomy by using BioGlue (CryoLife Europa Ltd, Hampshire, United Kingdom). This method struck us after we started using Bioglue for prevention of alveolar air leaks. The technique is fairly quick, simple and easy to reproduce. We presume others may have used it too but it does not appear to be described in the literature before.

## Technique

This fixation should be done after completing all the essential steps of resection such as testing of the bronchus, proper haemostasis, control of air leaks, and washing the pleural cavity. The anaesthetist is asked to inflate the lungs, the middle and lower lobe are inspected and any potential torsion corrected. A thin layer of Bioglue (CryoLife Europa Ltd, Hampshire, United Kingdom) is applied to the surface of middle lobe towards the oblique fissure and both lobes allowed to approximate for two minutes before any further manipulations. The Bioglue settles quickly and results in secure adherence of two lobes. The remaining Bioglue can be applied at the edges of lobes in oblique fissure with standard nozzle shaped tip as previously described to control air leaks [4]. This application is facilitated greatly by the new spatulated tip available with Bioglue. Chest drain can be inserted before or after the Bioglue application.

We have used this in five patients after right upper lobectomy to fix very mobile right middle lobe to right lower lobe and one left video assisted thoracoscopic (VAT) lower basal segmentectomy to anchor the apical segment of the lower lobe to the left upper lobe. In all cases we have found it really simple, quick and easily reproducible with good results. There was no complication related to Bioglue application.

## Discussion

Epplen and Jacobson (1930) [5] were the first group to describe pulmonary lobar torsion. Historically the right middle lobe is the most commonly affected lobe but other lobes have also shown to be affected. The relatively long bronchus and pulmonary artery has been postulated the cause of torsion on left side. Division of pulmonary ligament to facilitate expansion of remaining lung and for mediastinal lymph node dissection has been implicated as predisposing factors. Other predisposing factors are pneumothorax, pleural collections, and non-expanded lung after resection and single trunk pulmonary veins leading to a long narrow broncho-vascular pedicle. Apart from lung resection, lung torsion has also been reported with blunt chest trauma, accessory upper lobe, pneumonia, pleural effusion and pneumothorax.

A very high morbidity and mortality has been reported after torsion of lung [2]. Suturing (unsupported or reinforced) or stapling of middle and lower lobe and pleural flaps are few of the techniques described in the literature [3]. Suturing or stapling can result in alveolar air leaks resulting in prolonged need for chest drains, increased hospital stay, risk of infection and adds the cost of staplers. It also runs the risk of cutting through. Pleural flap creation is not easy to reproduce, adds one more surgical step and has a small risk of bleeding.

We have used this technique both in open and VATS lung resection and found it simple and reproducible. No serious complication has been described in literature with pulmonary use of BioGlue [4]. There are some fears of passage of blood born infection related to bovine product and infection in chest cavity because of foreign body implantation and slow absorption. We have not come across any report documenting such complication with BioGlue. In our limited experience we have not come across infection after Bioglue application. We always apply the glue on inflated lungs fearing unintentional restriction of lung expansion.

From our small but successful experience, we recommend this simple and quick technique for pulmonary fixation after segmentectomy or lobectomy to prevent potential torsion.

## Authors' contributions

The new idea was of JZ, he used the technique, MP as his registrar witness it, put the article together. Both authors read and approved the final manuscript.

## References

[B1] Cable DG, Deschamps C, Allen MS, Miller DL, Nichols FC, Trastek VF, Pairolero PC (2001). Lobar torsion after pulmonary resection: Presentation and outcome. J Thorac Cardiovasc Surg.

[B2] Wong PS, Goldstraw P (1992). Pulmonary torsion: A questionnaire survey and a survey of the literature. Ann Thorac Surg.

[B3] Kutlu CA, Olgac G (2006). Pleural flap to prevent lobar torsion: a novel technique. Eur J Cardiothorac Surg.

[B4] Epplen F, Jacobson AL (1930). Twisted pedicle of accessory lobe of the lung. JAMA.

[B5] Tansley P, Al-Mulhim F, Lim E, Ladas G, Goldstraw P (2006). A prospective, randomized, controlled trial of the effectiveness of BioGlue in treating alveolar air leaks. J Thorac Cardiovasc Surg.

